# Characteristic and phylogenetic analyses of chloroplast genome for *Syringa komarowii* C.K.Schneid. (Oleaceae) from Huoditang, China, an important horticultural plant

**DOI:** 10.1080/23802359.2021.1918029

**Published:** 2021-04-27

**Authors:** Yong-Qin Cheng, Zai-Min Jiang, Jing Cai

**Affiliations:** a College of Forestry, Northwest A&F University, Yangling, China; b College of Life Science, Northwest A&F University, Yangling, China; c Qinling National Forest Ecosystem Research Station, Ningshan, China

**Keywords:** Complete chloroplast genome, phylogenetic analysis, *S. komarowii*

## Abstract

The genus *Syringa* (Oleaceae) comprises some of the most important cultivated horticultural trees worldwide. *Syringa komarowii*, is one of these important horticultural plants. We sequenced the complete chloroplast (cp) genomes of *S. komarowii* (previously identified as var. *reflexa*) using Illumina Hiseq X Ten platform. The cp genome exhibits a typical quadripartite structure with 158,020 bp in length, which consists of two copies of inverted repeat (IR) regions (25,676 bp) separated by a large single copy (LSC) region (87,628 bp) and a small single copy (SSC) region (19,040 bp). The cp genome of *S. komarowii* encodes a total of 132 genes, including 88 protein-coding genes, 36 tRNA genes and 8 rRNA genes. A maximum likelihood (ML) phylogenetic analysis resolved that *S. komarowii* in a clade with *S. wolfii* and *S. yunnanensis*. The ML tree also showed *Syringa* appeared more closely related to *Ligustrum* than to the other genera in the Oleaceae.

*Syringa* is distributed in China, North Korea, Japan and Southeast Europe. The species of *Syringa* have high ornamental, medicinal, economic and environmental value, and are an indispensable landscape ornamental plant. China has most of the wild resources of *Syringa* and is recognized as the natural distribution center for *Syringa* (Zang and Cui 2000). *Syringa. komarowii*, arguably has the best ornamental values in the genus *Syringa*, because of its contrasting pink outer corolla and white inner corolla. In this study, the complete chloroplast (cp) genome of *S. komarowii*, previously accepted with varietal status as *S. komarowii* var. *reflexa*, is reported to provide genomic data for future research.

*Syringa komarowii* var. *reflexa* was collected from Huoditang (located in the center of Qinling Mountains, is the teaching and experimental forest farm of Northwest A&F University) (36.32°N, 108.35°E). The samples were dried with silica gel and stored in the herbarium of Northwest A&F University (email: courage4132231@163.com, Chaoyang Zhang) under the voucher number SY201913. DNA was extracted from leaf tissue using the Plant Genomic DNA kit (Tiangen Biotech, Beijing, China) following the modified Cetyl Trimethyl Ammonium Bromide method (Doyle 1987). The complete genome sequencing was performed using the Illumina HiSeq X Ten Platform (Illumina, San Diego, CA) with 350 bp pair-end reads on at Biomarker Technologies Inc. (Beijing, China). The high-quality paired-end reads were trimmed and assembled with MIRA version 4.0.2 (Chevreux et al. 2004) and MITObim-master version1.7 (Hahn et al. 2013) using the *S. yunnanensis* (Olofsson et al. 2019) chloroplast genome sequence as a reference (GenBank number NC_042468). Annotations were performed using Geneious R version 10.2.2 (Biomatters, Auckland, New Zealand). The chloroplast genome sequence was deposited in GenBank, accession number MT648823.

The cp genome of *S. komarowii* is 158,020 bp in length and contains one LSC of 87,628 bp and one SSC of 19,040 bp, which were separated by a pair of IRs of 25,676 bp. The guanine and cytosine content of the cp genome, LSC, SSC and IR regions were 37.9%, 36.2%, 32.6%, and 43.0%, respectively. In total, 132 genes were predicted in the plastid genomes of *S. komarowii*, including 8 rRNA, 36 tRNA, and 88 protein-coding genes. Among these genes, 14 genes (*atpF*, *ndhA*, *ndhB*, *petB*, *petD*, *rpl2*, *rps12*, *rps16*, *rpoC1*, *trnI-GAU*, *trnA-UGC*, *trnK-UUU*, *trnG-GCC*, and *trnL-UAA*,) have one intron, and two genes (*ycf3* and *clpP*) contain two introns. The longest intron was *trnK-UUU* at 2,494 bp.

Phylogenetic trees were reconstructed using Maximum likelihood (ML) methods with cp genome sequences of 53 species. *Scrophularia henryi* was set as an outgroup to infer the phylogenetic positions of *S. komarowii* within *Syringa* and relationships of Oleaceae. All of the 53 cp genome sequences were aligned using MAFFT (Katoh and Standley 2013) with default parameter. To reconstruct the phylogenetic relations of these species, we used IQ-TREE 1.6.12 (Nguyen et al. 2015) with ML analysis, using the general time reversible (GTR) model by the program jModeltest version 2.1.7 (Darriba et al. 2012). The node stability was assessed using a rapid bootstrapping analysis with 1000 replicates. The phylogenetic tree indicated that *S. komarowii* was close to *S. wolfii* and *S. yunnanensis* (MLBS = 100%), *Syringa* closely related to *Ligustrum* (Figure 1), which is consistent with previous molecular results (Li et al. 2002). The cp genome of *S. komarowii* provides a valuable resource for evolutionary research in the Oleaceae and also demonstrates the effectiveness of chloroplast phylogenomics to further resolve the phylogenetic relationships in the family.

**Figure 1.**
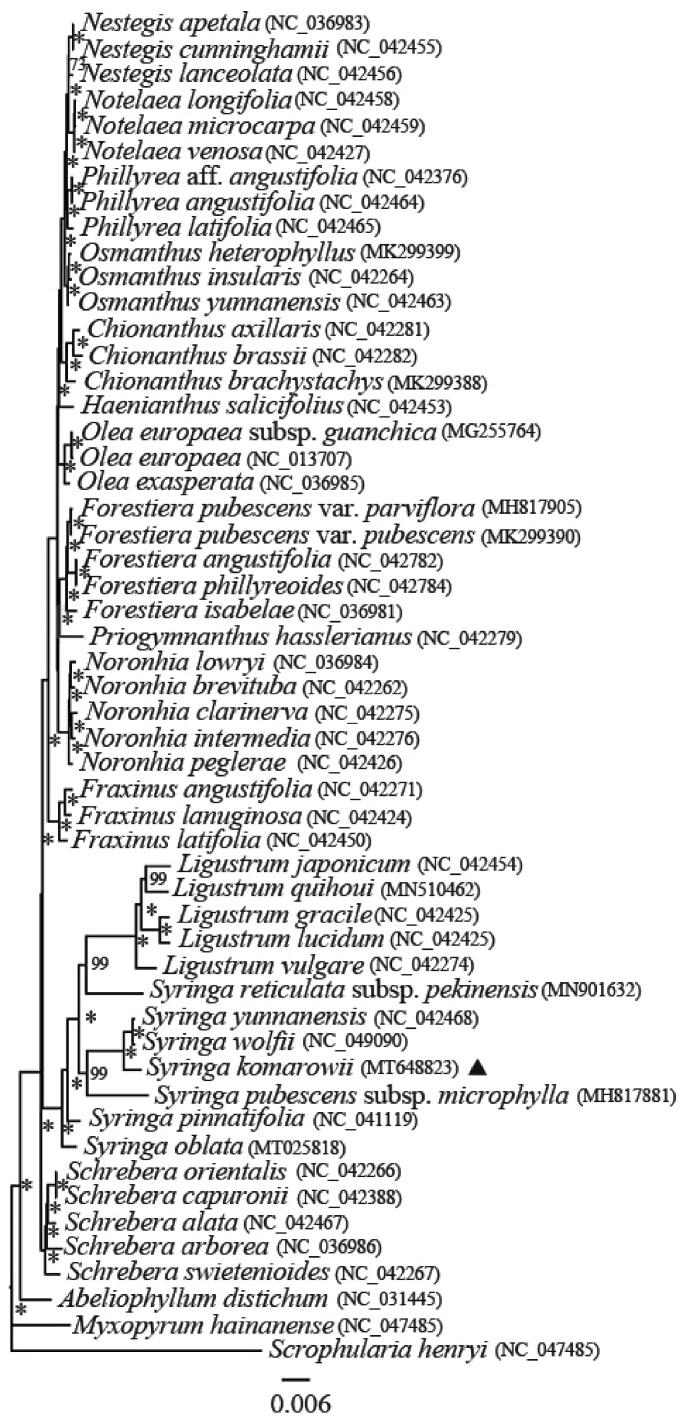
Maximum likelihood phylogenetic tree inferred in Iqtree from the alignment of 53 cp genome sequences. Node support was evaluated with 1000 bootstrap replicates and is indicated near branches (* = 100%).

## Data Availability

The genome sequence data that support the findings of this study are openly available in GenBank of NCBI at (https://www.ncbi.nlm.nih.gov/) under the accession no. MT648823. The associated BioProject, SRA, and Bio-Sample numbers are PRJNA705853, SRR13823815, and SAMN18104659 respectively.

## References

[CIT0001] Chevreux B, Pfisterer T, Drescher B, Driesel AJ, Müller WE, Wetter T, Suhai S. 2004. Using the miraEST assembler for reliable and automated mRNA transcript assembly and SNP detection in sequenced ESTs. Genome Res. 14(6):1147–1159. 15140833 10.1101/gr.1917404 PMC419793

[CIT0002] Darriba D, Taboada GL, Doallo R, Posada D. 2012. jModelTest 2: more models, new heuristics and parallel computing. Nat Methods. 9(8):772–772. 10.1038/nmeth.2109 PMC4594756 22847109

[CIT0003] Doyle JJ. 1987. A rapid DNA isolation procedure for small quantities of fresh leaf tissue. Phytochem Bull. 19:11–15.

[CIT0004] Hahn C, Bachmann L, Chevreux B. 2013. Reconstructing mitochondrial genomes directly from genomic next-generation sequencing reads - a baiting and iterative mapping approach. Nucleic Acids Res. 41(13):e129–e129. 23661685 10.1093/nar/gkt371 PMC3711436

[CIT0005] Katoh K, Standley DM. 2013. MAFFT multiple sequence alignment software version 7: improvements in performance and usability. Mol Biol Evol. 30(4):772–780. 23329690 10.1093/molbev/mst010 PMC3603318

[CIT0006] Li JH, Alexander JH, Zhang DL. 2002. Paraphyletic *Syringa* (Oleaceae): Evidence from sequences of nuclear ribosomal DNA ITS and ETS regions. Syst Bot. 27:592–597.

[CIT0007] Nguyen LT, Schmidt HA, von Haeseler A, Minh BQ. 2015. IQ-TREE: a fast and effective stochastic algorithm for estimating Maximum-likelihood phylogenies. Mol Biol Evol. 32(1):268–274. 25371430 10.1093/molbev/msu300 PMC4271533

[CIT0008] Olofsson JK, Cantera I, Van de Paer C, Hong‐Wa C, Zedane L, Dunning LT, Alberti A, Christin P‐A, Besnard G. 2019. Phylogenomics using low-depth whole genome sequencing: a case study with the olive tribe. Mol Ecol Resour. 19(4):877–892. 30934146 10.1111/1755-0998.13016

[CIT0009] Zang SY, Cui HX. 2000. Syringa. Shanghai: Shanghai Scientific and Technical Publishers; p. 1–5.

